# Aloe & Hernstein

**Published:** 1878-10-20

**Authors:** 


ALOE & HERNSTEIN. This splended establishment, Importers and Manufactures of surgical instruments; trusses, supports, braces etc. etc., renew thier advertisement in the Record as may be seen on coloured pages

Their improved Physicians Saddle Bags have met with great favor to our knowledge. We cordially recommend this firm to the patronage of our readers.

